# A Convenient Synthesis of Diketopyrrolopyrrole Dyes

**DOI:** 10.3390/molecules26164758

**Published:** 2021-08-06

**Authors:** Vitor A. S. Almodôvar, Augusto C. Tomé

**Affiliations:** LAQV-REQUIMTE, Department of Chemistry, University of Aveiro, 3810-193 Aveiro, Portugal; v.almodovar@ua.pt

**Keywords:** diketopyrrolopyrrole, Suzuki–Miyaura reaction, fluorescence, Ferrari red

## Abstract

Diketopyrrolo[3,4-*c*]pyrroles (DPP) are high-performance organic optoelectronic materials. They have applications in solar cells, fluorescent probes, bioimaging, photodynamic/photothermal therapy, and in many other areas. This article reports a convenient two-step synthesis of various DPP dyes from Pigment Red 254, an inexpensive commercial pigment. The synthesis includes a Suzuki–Miyaura cross-coupling reaction of a bis(4-chlorophenyl)DPP derivative with aryl and hetaryl boronic acids under mild reaction conditions. The new dyes show large Stokes shifts and high fluorescence quantum yields, important features for their potential use in technical and biological applications.

## 1. Introduction

3,6-Diaryl-2,5-dihydropyrrolo[3,4-*c*]pyrrole-1,4-diones (also known as diketopyrrolopyrroles, DPP) are a class of brilliant and strongly fluorescent pigments and dyes. These high-performance compounds gained wide attention in recent years due their outstanding properties, namely large extinction coefficients, high fluorescent quantum yields, and environment and heat stability, which make them excellent candidates for a range of technical and biological applications [1]. In fact, DPP derivatives have applications in materials technology, from paint pigments to dye-sensitized solar cells (DSSC) [2,3,4,5,6], organic solar cells [7,8,9,10,11,12,13], organic electronics [14,15,16,17], fluorescent probes [18,19,20,21,22,23], materials for lithium-ion batteries [24], for ionic charge storage [25], or for the removal of micropollutants from water [26], etc. Besides that, the studies concerning their potential use in biological applications, namely as antibacterial agents [27,28,29], in photodynamic/photothermal therapy [30,31,32,33,34,35,36,37,38], or bioimaging/theranostics [39,40,41,42,43], have increased in recent years. 

The DPP bicyclic system is usually generated from the reaction of aryl nitriles with diethyl (or diisopropyl) succinate in the presence of a strong base, and frequently the expected symmetrical DPP derivatives are obtained in low to moderate yields [44]. Other approaches, developed for the synthesis of non-symmetrical DPP derivatives, require precursors not easily available or long synthetic routes [1,45,46,47]. An attractive approach to new DPPs involves the modification of adequately functionalized DPP derivatives. The Suzuki–Miyaura, Stille, Sonogashira, and Heck coupling reactions using bromophenyl, bromothienyl, and bromofuryl DPP derivatives have been extensively used for the preparation of small DPP molecules and polymeric DPP-based materials [16,17,26,48,49,50,51,52,53]. Although these are very versatile and efficient reactions, again, the required brominated derivatives are expensive or not easily accessible. In this paper we report a simple procedure for the synthesis of DPP derivatives bearing functional groups adequate for further transformations starting from an inexpensive commercial DPP pigment.

## 2. Results and Discussion

### 2.1. Design and Synthesis 

Pigment Red 254 (also known as Ferrari red) is an inexpensive commercial DPP pigment. However, it has scarcely been used as precursor to other DPP derivatives and the reported transformations are mainly *N*-alkylations [54,55,56,57]. Although it contains two 4-chlorophenyl groups that can be used to get access to other DPP derivatives by direct substitution of the chlorine atoms, this approach has been rarely used [28,58,59,60]. Here we report that this pigment can be successfully converted into other DPP derivatives, with adequate functional groups for further transformations, by Suzuki–Miyaura cross-coupling reaction with aryl and hetaryl boronic acids. This method is an excellent alternative to the previously reported one that requires the synthesis of the corresponding diboronate followed by reaction with iodoarenes, with both steps requiring Pd catalysis [28,60]. This new route involves only one Pd catalyzed step, requires mild conditions, and provides the new compounds in higher yields.

**Scheme 1:**
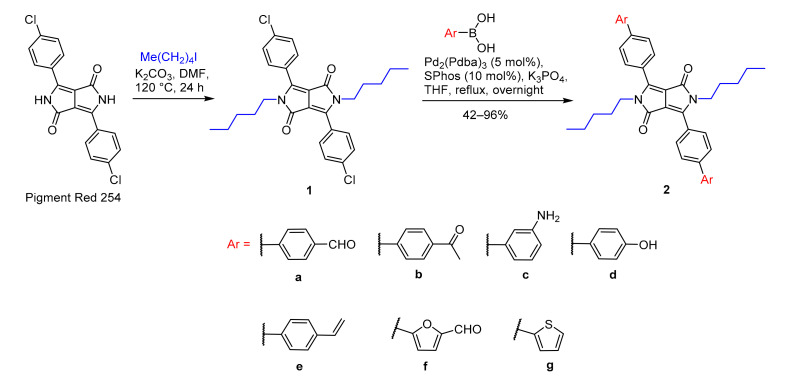
Synthesis of DPP dyes **2a**–**g**.

The synthetic procedures to obtain the DPP derivatives are summarized in Scheme 1. The first step involved the *N*,*N*’-dialkylation of Pigment Red 254 with 1-iodopentane as previously reported [28]. This step is essential to convert the pigment (insoluble) into a dye (soluble), thus facilitating the following reactions and the purification process of the resulting products. Dye **1**, which is soluble in most common organic solvents, was then used in Suzuki–Miyaura cross-coupling reactions with various boronic acid derivatives affording dyes **2a**–**g** in moderate to excellent yields (42–96%). This method is compatible with the presence of a diversity of functional groups, namely formyl, acetyl, amino, hydroxy, vinyl, and others not shown here, that may be used for further transformations as already shown by us [28]. Comparing the yield for compound **2a** by the previous method (42% overall yield from **1**) [28] with the one reported here (80% yield), it is evident that this new approach affords the expected DPP derivatives in much higher yields.

### 2.2. Structural and Photophysical Characterization 

The structures of dyes **2a**–**g** were unambiguously established from their ^1^H and ^13^C NMR and mass spectra. Their absorption and emission spectra were also obtained (see Supplementary Materials). As expected, the ^1^H NMR spectra of all compounds show the resonance of the *N*-CH_2_ protons as a triplet at *ca*. 3.8 ppm and the signals of the remaining protons of pentyl groups between 0.75–1.75 ppm. The signals of the *p*-substituted phenyl rings linked to the DPP core appear as AB systems centered at *ca*. 7.9 ppm, and the signals of the protons of the peripheric aryl/hetaryl groups appear at slightly higher fields (between 6.75 and 7.75 ppm). In addition, for compounds **2a**, **2b**, and **2f**, the ^1^H NMR spectra show the diagnostic signals corresponding to the formyl and acetyl groups at 10.08, 2.66, and 9.71 ppm, respectively. The ^13^C NMR spectra of compounds **2a**–**g** are also consistent with the proposed structures, showing all the expected peaks. The mass spectra of compounds **2a**–**g** show, in all cases, the protonated molecular ion [M + H]^+^ as the base peak.

**Figure 1:**
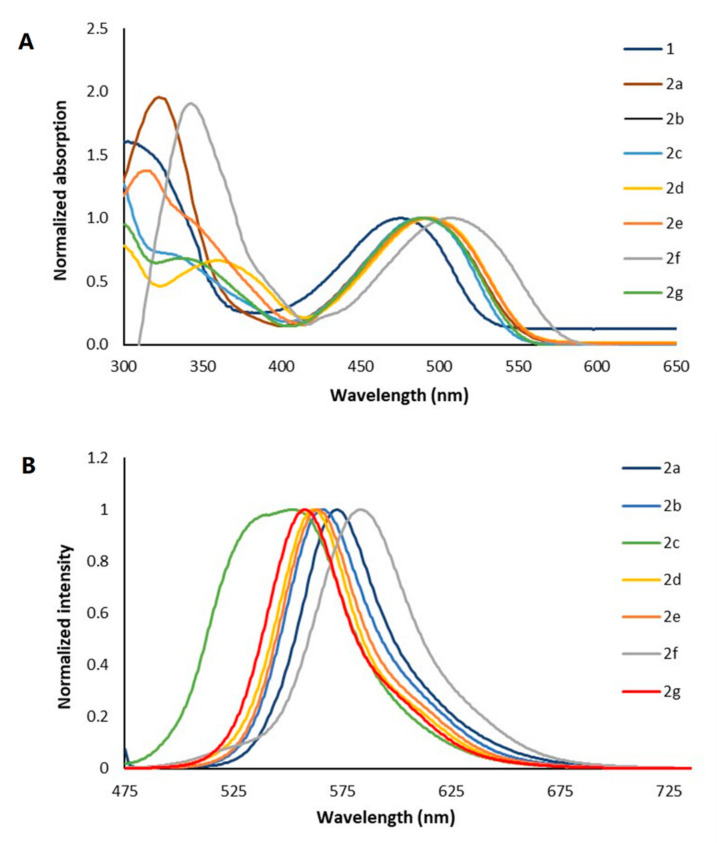
Normalized (**A**) absorption and (**B**) fluorescence spectra of **2a**–**g** in DMF.

The UV–Vis and fluorescence spectra of compounds **2a**–**g** in DMF are shown in Figure 1 and their photophysical properties are summarized in Table 1. Compared with DPP **1** (λ_max_ = 476 nm), the λ_max_ for all dyes are bathochromically shifted, with the largest shift observed for the furan derivative **2f**. Stokes shifts for dyes **2** were typically in the range of 68 and 73 nm, as observed for other 3,6-bis(biphenyl)DPP derivatives [49]. However, due to the presence of strong electron withdrawing and donating groups, compounds **2a** and **2c** show Stokes shifts of 83 and 54 nm, respectively. The fluorescence quantum yield (*Ø*_F_) for each dye is also shown in Table 1. Considering those values, dyes **2** can be divided into two groups: those with electron withdrawing groups or heterocyclic rings (**2a**, **2b**, **2f**, and **2g**) have *Ø*_F_ in the range 0.8–0.9, while compounds **2c**, **2d**, and **2e**, bearing electron donating groups, have *Ø*_F_ near 0.4.

As highlighted in the introduction, the use of DPP derivatives in a large diversity of applications is being actively studied. In this context, considering the fluorescence properties displayed by dyes **2**, they, or their adequately functionalized derivatives, are potentially useful compounds as molecular probes for bioimaging/theranostics.

## 3. Materials and Methods

### 3.1. Chemicals and Instrumentation

The reagents used in this work were purchased from Merck Life Science (Algés, Portugal) and were used as received. The solvents were used as received or distilled and dried by standard procedures. Analytical thin-layer chromatography (TLC) was carried out on precoated sheets with silica gel (Merck 60, 0.2 mm thick). Preparative thin-layer chromatography was carried out on 20 cm × 20 cm glass plates precoated with a layer of silica gel 60 (0.5 mm thick) and activated in an oven at 100 ℃ for 12 h. ^1^H and ^13^C NMR spectra were recorded on a Bruker Avance 300 or Bruker Avance 500. CDCl_3_ was used as a solvent and tetramethylsilane (TMS) as an internal reference. The chemical shifts are expressed in δ (ppm) and the coupling constants (*J*) in hertz (Hz). UV–Vis spectra were recorded on a Shimadzu UV-2501PC spectrophotometer using DMF as the solvent. The emission spectra were recorded with a Jasco FP-8300 spectrofluorometer using DMF as the solvent. Mass spectra were recorded using a Micromass Q-TOF-2TM mass spectrometer and CHCl_3_ as the solvent. Fluorescence quantum yields (*Ø*_F_) were calculated using fluorescein as a reference (*Ø*_F_ = 0.55 in DMF) [61]. Melting points were determined with a Büchi B-540 apparatus.

### 3.2. Synthesis

#### General Procedure for the Suzuki–Miyaura Cross-Coupling Reactions

To a suspension of K_3_PO_4_ (4 equiv.) in degassed THF (20.0 mL), DPP **1** (80 mg, 0.161 mmol), the corresponding boronic acid (0.644 mmol, 4 equiv.) and catalytic amounts of Pd_2_(Pdba)_3_ (5 mol%) and SPhos (10 mol%) were added. The resulting mixture was refluxed overnight under a nitrogen atmosphere. It was then cooled down to room temperature and the solvent was removed under reduced pressure. The products were purified by preparative TLC using mixtures of dichloromethane/hexane or ethyl acetate/hexane as eluents. For the synthesis of **2d**, Pd(OAc)_2_ was used as a catalyst and butan-1-ol as a solvent.

*4*′*,4‴-(3,6-Dioxo-2,5-dipentyl-2,3,5,6-tetrahydropyrrolo[3,4-c]pyrrole-1,4-diyl)bis([1,1*′*-biphenyl]-4-carbaldehyde)* (**2a**): 82 mg, 80% yield; mp: 177.4–179.2 ℃; ^1^H NMR (300 Hz, CDCl_3_) δ (ppm), 10.08 (s, 2H), 8.01–7.94 (m, 8H), 7.83–7.79 (m, 8H), 3.81 (t, *J* = 7.6 Hz, 4H), 1.70–1.62 (m, 4H), 1.28–1.22 (m, 8H), 0.83 (t, *J* = 6.7 Hz, 6H); ^13^C NMR (CDCl_3_, 75 MHz) δ (ppm) 191.8, 162.8, 147.9, 145.8, 142.3, 135.8, 130.5, 129.4, 128.2, 127.9, 127.8, 110.2, 42.1, 29.3, 28.9, 22.2, 13.9; MS (ESI^+^) *m*/*z*: 637.4 ([M + H]^+^, 100%).

*3,6-Bis(4*′*-acetyl-[1,1*′*-biphenyl]-4-yl)-2,5-dipentyl-2,5-dihydropyrrolo[3,4-c]pyrrole-1,4-dione* (**2b**): 82 mg, 77% yield; mp: 279.8–280.4 ℃; ^1^H NMR (CDCl_3_, 300 MHz), δ (ppm): 8.08 (d, *J* = 8 Hz, 4H), 7.97 (d, *J* = 9 Hz, 4H), 7.83–7.75 (m, 8H) 3.82 (t, *J* = 7.5 Hz, 4H), 2.66 (s, 6H), 1.69–1.64 (m, 4H), 1.30–1.25 (m, 8H), 0.85 (t, *J* = 7 Hz, 6H); ^13^C NMR (CDCl_3_, 75 MHz) δ (ppm) 197.7, 162.8, 147.9, 144.4, 142.5, 136.5, 129.1, 129.0, 127.9, 127.5, 110.2, 42.1, 29.3, 28.9, 26.8, 22.2, 13.9; MS (ESI^+^) *m*/*z*: 665.6 ([M + H]^+^, 100%).

*3,6-Bis(3*′*-amino-[1,1*′*-biphenyl]-4-yl)-2,5-dipentyl-2,5-dihydropyrrolo[3,4-c]pyrrole-1,4-dione* (**2c**): 41 mg, 42% yield; mp: 233.5–235.1 ℃; ^1^H NMR (CDCl_3_, 300 MHz), δ (ppm): 7.90 (d, *J* = 8.6 Hz, 4H), 7.72 (d, *J* = 8.6 Hz, 4H), 7.26 (t, *J* = 8 Hz, 2H), 7.04 (ddd, *J* = 8, 2 and 1 Hz, 2H), 6.95 (t, *J* = 2 Hz, 2H), 6.72 (ddd*, J* = 8, 2, and 1 Hz, 2H), 3.80 (t, *J* = 7.5 Hz, 4H), 1.70–1.63 (m, 4H), 1.29–1.24 (m, 8H), 0.83 (t, *J* = 7.0 Hz, 6H); ^13^C NMR (CDCl_3_ + (CD_3_)_2_CO, 75 MHz), δ (ppm): 162.6, 149.4, 141.1, 130.2, 129.7, 127.5, 116.2, 115.1, 113.4, 41.8, 29.3, 29.1, 22.3, 13.8; MS (ESI^+^) *m*/*z*: 611.4 ([M + H]^+^, 100%).

*3,6-Bis(4*′*-hydroxy-[1,1*′*-biphenyl]-4-yl)-2,5-dipentyl-2,5-dihydropyrrolo[3,4-c]pyrrole-1,4-dione* (**2d**): (61 mg, 62% yield); mp: 316.7–317.2 ℃ (crystalized from CH_2_Cl_2_/MeOH); ^1^H NMR (DMSO, 300 MHz), δ (ppm): 9.81 (s, 2H), 7.92 (d, *J* = 8.5 Hz, 4H), 7.83 (d, *J* = 8.5 Hz, 4H), 7.65 (d, *J* = 8.6 Hz, 4H), 6.9 (d, *J* = 8.6 Hz, 2H), 3.77 (t, *J* = 7.5 Hz, 4H), 1.51–1.43 (m, 4H), 1.20–1.13 (m, 8H), 0.78 (t, *J* = 7 Hz, 6H); ^13^C NMR (DMSO, 75 MHz), δ (ppm): 162.1, 158.5, 147.9, 143.1, 129.9, 129.7, 128.5, 126.5, 126.1, 116.4, 109.1, 41.4, 28.8, 28.7, 21.9, 14.2; MS (ESI^+^) *m*/*z*: 643.5 ([M + H]^+^, 100%).

*2,5-Dipentyl-3,6-bis(4*′*-vinyl-[1,1*′*-biphenyl]-4-yl)-2,5-dihydropyrrolo[3,4-c]pyrrole-1,4-dione* (**2e**): 98 mg, 96% yield; mp: 233.4–235.1 ℃; ^1^H NMR (CDCl_3_, 300 MHz), δ (ppm): 7.93 (d, *J* = 9 Hz, 4H), 7.78 (d, *J* = 9 Hz, 4H), 7.64 (d, *J* = 9 Hz, 4H), 7.53 (d, *J* = 9 Hz, 4H), 6.83–6.73 (m, 2H), 5.83 (d, *J* = 12 Hz, 2H), 5.32 (d, *J* = 12 Hz, 2H), 3.82 (t, *J* = 7.5 Hz, 4H), 1.72–1.63 (m, 4H), 1.31–1.25 (m, 8H), 0.85 (t, *J* = 7 Hz, 6H); ^13^C NMR (CDCl_3_, 75 MHz), δ (ppm): 162.9, 148.1, 143.2, 139.3, 137.4, 136.3, 129.3, 127.3, 127.1, 126.9, 114.5, 109.9, 42.1, 29.3, 28.9, 22.2, 13.9; MS (ESI^+^) *m*/*z*: 633.6 ([M + H]^+^, 100%).

*5,5*′*-((3,6-Dioxo-2,5-dipentyl-2,3,5,6-tetrahydropyrrolo[3,4-c]pyrrole-1,4-diyl)bis(4,1-phenylene))bis(furan-2-carbaldehyde)* (**2f**): 63 mg, 63% yield; mp: 233.4–235.1 ℃. ^1^H NMR (CDCl_3_, 300 MHz), δ (ppm): 9.71 (s, 2H), 8.0 (d, *J* = 9 Hz, 4H), 7.95 (d, *J* = 9 Hz, 4H), 7.36 (d, *J* = 3 Hz, 2H), 6.98 (d, *J* = 6 Hz, 2H), 3.79 (t, *J* = 7.5 Hz, 4H), 1.66–1.61 (m, 4H), 1.28–1.24 (m, 8H), 0.85 (t, *J* = 6.9 Hz, 6H); ^13^C NMR (CDCl_3_, 75 MHz), δ (ppm): 177.5, 162.6, 158.0, 152.5, 147.6, 141.2, 131.2, 129.3, 128.9, 125.6, 109.3, 42.2, 29.7, 28.9, 22.2, 13.9; MS (ESI^+^) *m*/*z*: 617.5 ([M + H]^+^, 100%).

*2,5-Dipentyl-3,6-bis(4-(thiophen-2-yl)phenyl)-2,5-dihydropyrrolo[3,4-c]pyrrole-1,4-dione* (**2g**): 70 mg, 73% yield; mp: 170.3–172.1 ℃. ^1^H NMR (CDCl_3_, 300 MHz), δ (ppm): 7.90 (d, *J* = 9 Hz, 4H), 7.77 (d, *J* = 9 Hz, 4H), 7.58–7.60 (m, 2H), 7.47–7.42 (m, 4H), 3.81 (t, *J* = 7.5 Hz, 4H), 1.69–1.61 (m, 4H), 1.30–1.24 (m, 8H), 0.85 (t, *J* = 7.5 Hz, 6H); ^13^C NMR (CDCl_3_, 75 MHz), δ (ppm): 162.9, 147.9, 141.3, 138.4, 137.2, 129.9, 126.8, 126.1, 121.8, 109.9, 42.1, 29.3, 28.9, 22.2, 13.9; MS (ESI^+^) *m*/*z*: 593.4 ([M + H]^+^, 100%).

## 4. Conclusions

Pigment Red 254 was used as starting material to prepare, in two steps only, adequately functionalized DPP derivatives. The resulting compounds bear a range of functional groups that may be used for further transformations, namely for the introduction of functional units with specific physical/electronic properties or biological functions. The seven compounds reported here show large Stokes shifts and high fluorescence quantum yields, important features for their potential application in various fields. 

## Figures and Tables

**Table 1:** Spectroscopic data for compounds **2a**–**g** ^a^.

Compound	λ_max_ (nm) Absorption	ε (M^–1^ cm^–1^)	λ_max_ (nm) Emission	Stokes Shift (nm)	*Ø*_F_ (DMF)
**2a** [28]	492	22,265	575	83	0.87
**2b**	493	11,406	566	73	0.90
**2c**	498	14,107	552	54	0.40
**2d**	494	11,634	562	68	0.41
**2e**	493	33,694	563	70	0.39
**2f**	509	47,254	582	73	0.88
**2g**	489	35,735	560	71	0.81

^a^ All measurements were obtained in DMF solutions (between 1 × 10^−6^ M and 5 × 10^−5^ M) of each compound.

## Data Availability

The data presented in this study are available in supplementary material.

## Supplementary Materials

The following are available online: ^1^H and ^13^C NMR spectra, and absorption and emission spectra.

## Sample Availability

Samples of compounds **2a**–**g** are available from the authors.
