# Supplementary material for: A Convenient Synthesis of Diketopyrrolopyrrole Dyes
Source: Molecules. 2021 Aug 6;26(16):4758. doi: 10.3390/molecules26164758 (PMC8401603; doi:10.3390/molecules26164758)

Supporting Information for:

# A convenient synthesis of diketopyrrolopyrrole dyes

Vitor A. S. Almodovar and Augusto C. Tomé *

*LAQV-REQUIMTE, Department of Chemistry, University of Aveiro, 3810-193 Aveiro, Portugal*

## $^{1}$H NMR, $^{13}$C NMR, absorption and emission spectra of compounds 2a–2g

## Compound 2a

### $^{1}$H NMR spectrum of 2a

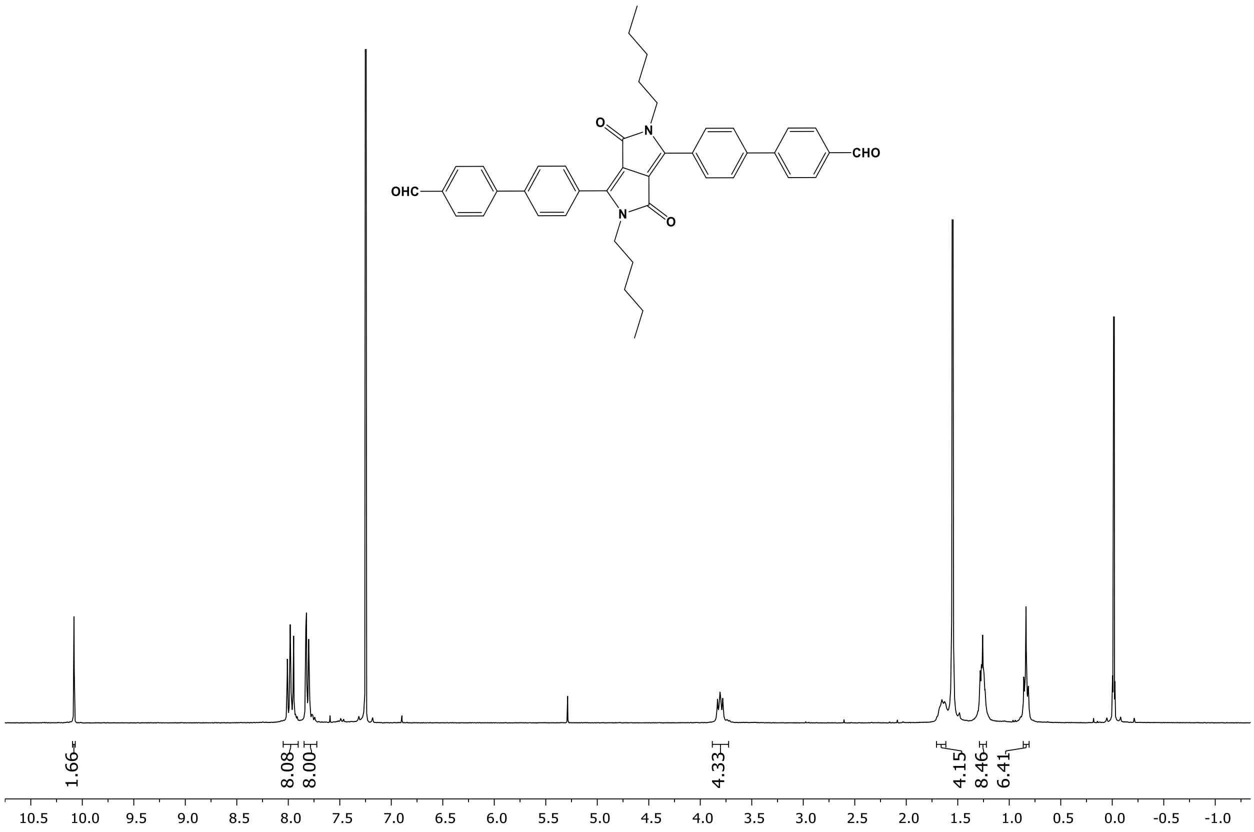

$^{13}C$ NMR spectrum of 2a

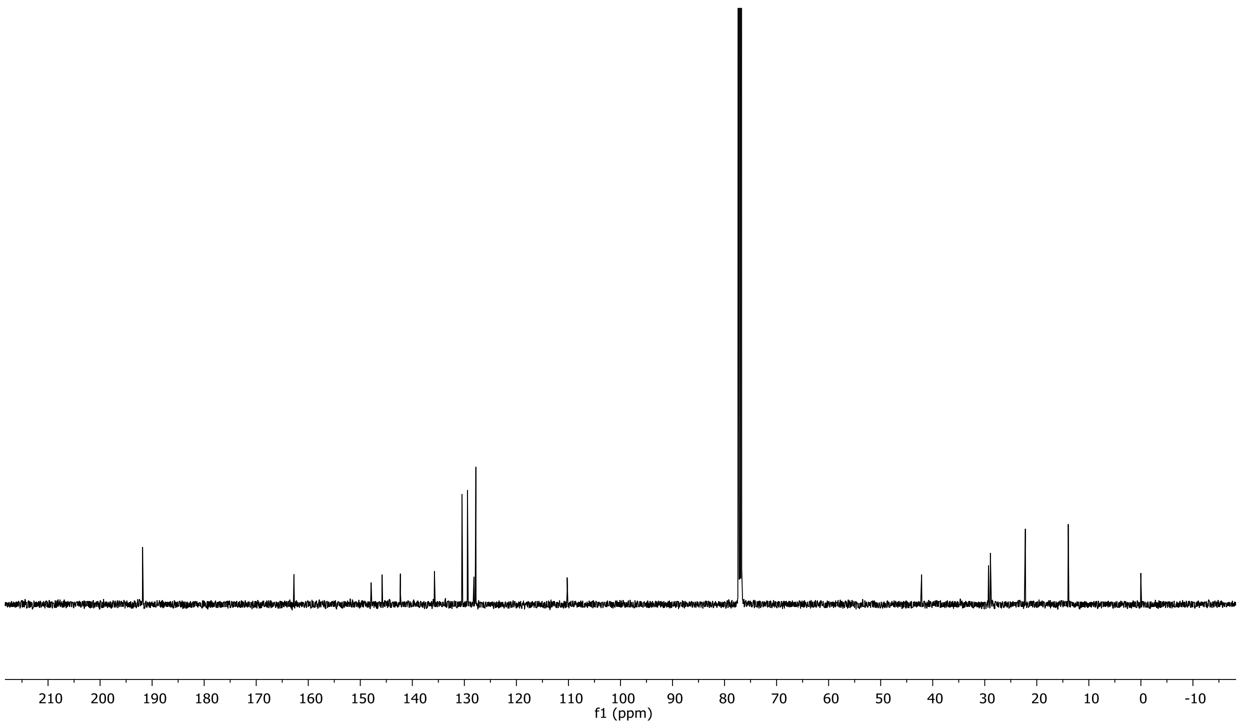


Absorption and emission spectra of 2a

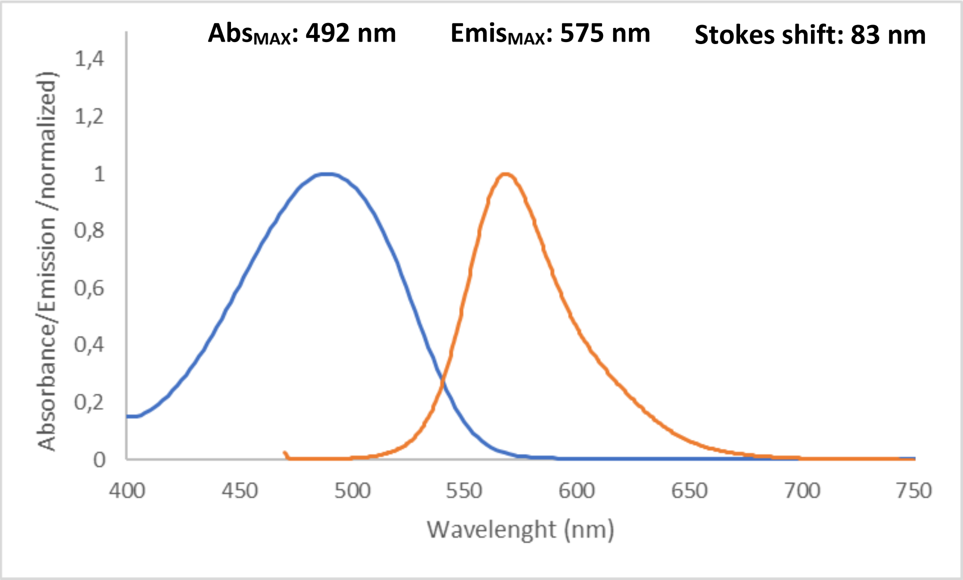

# Compound 2b

## $^{1}$H NMR spectrum of 2b

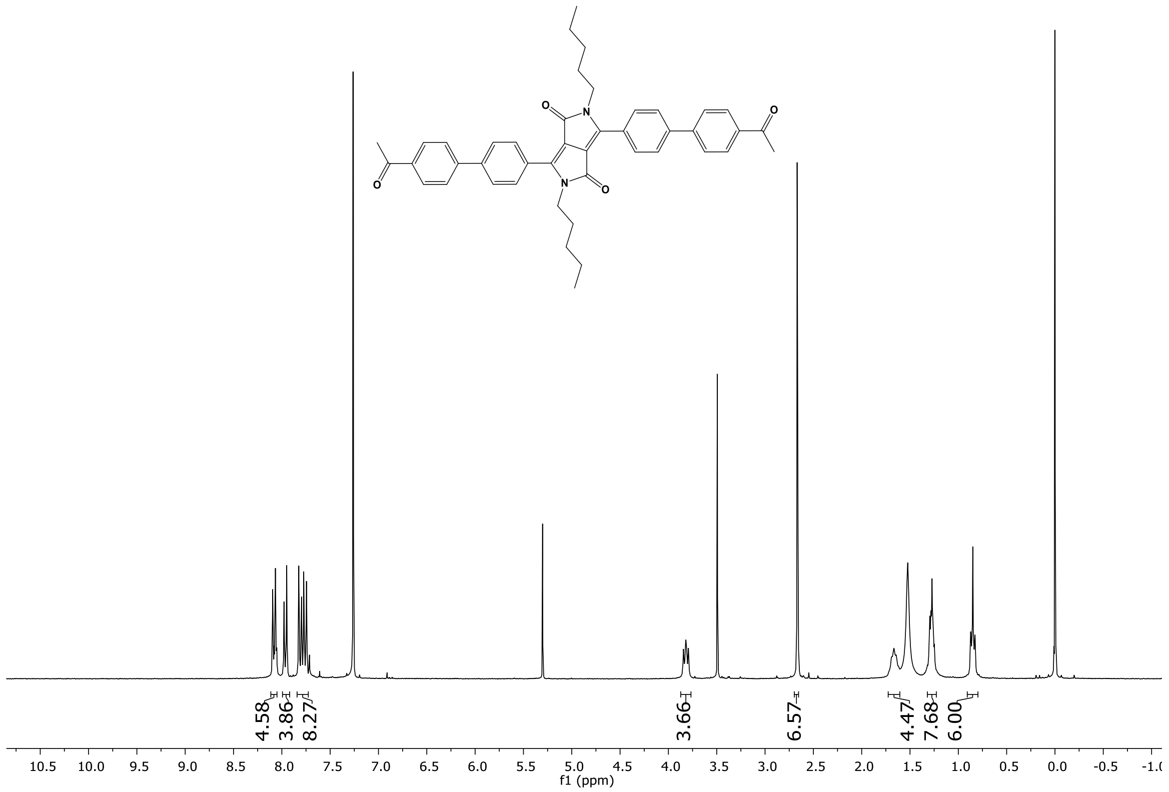


## $^{13}$C NMR spectrum of 2b

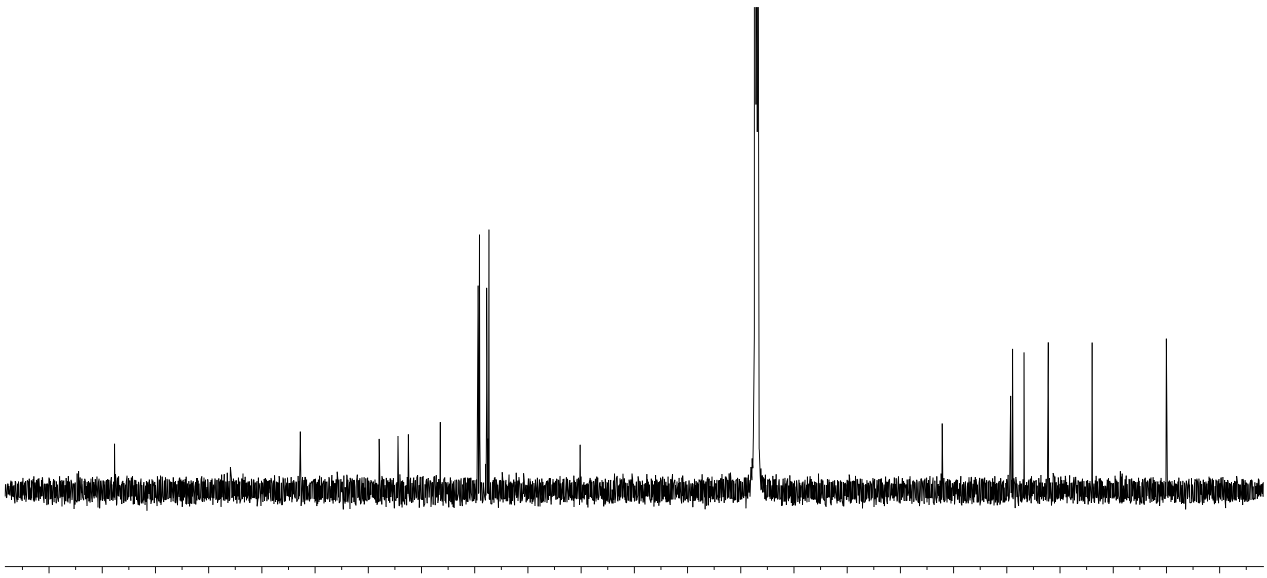

**Absorption and emission spectra of 2b**

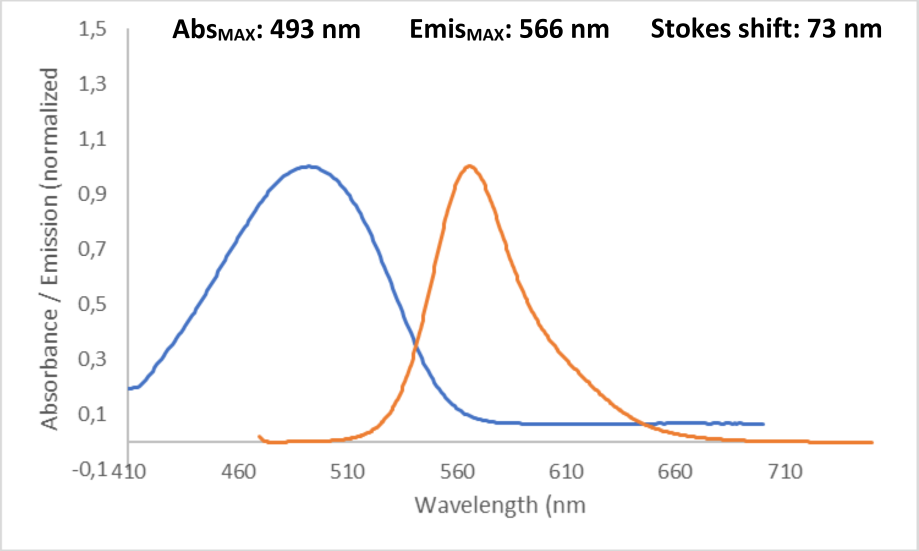

# Compound 2c

## $^{1}$H NMR spectrum of 2c

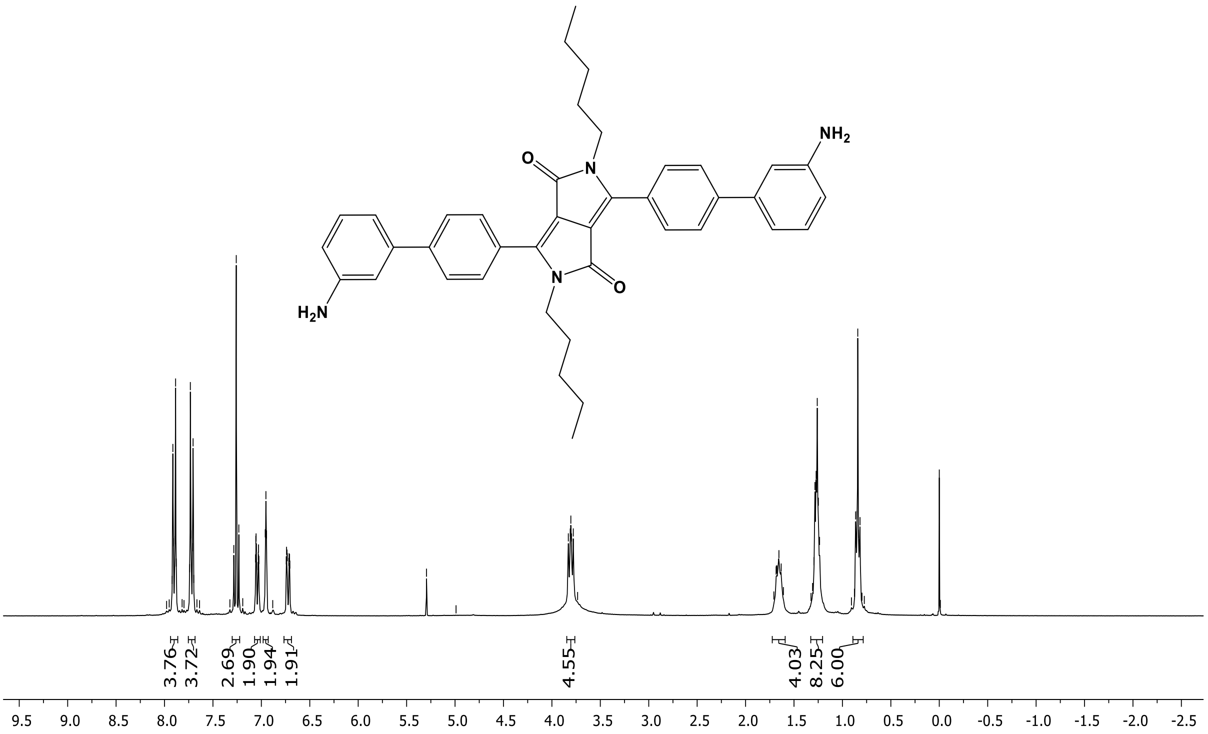


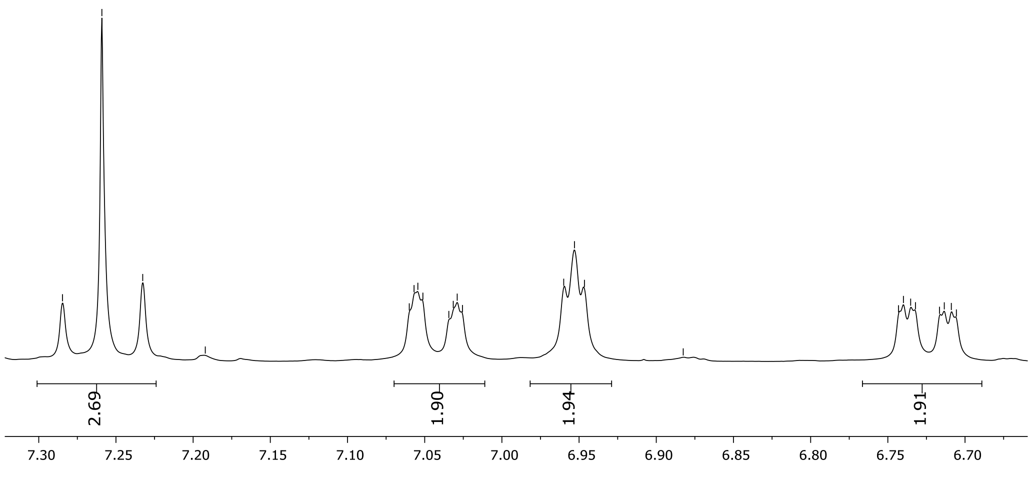

$^{13}$C NMR spectrum of 2c

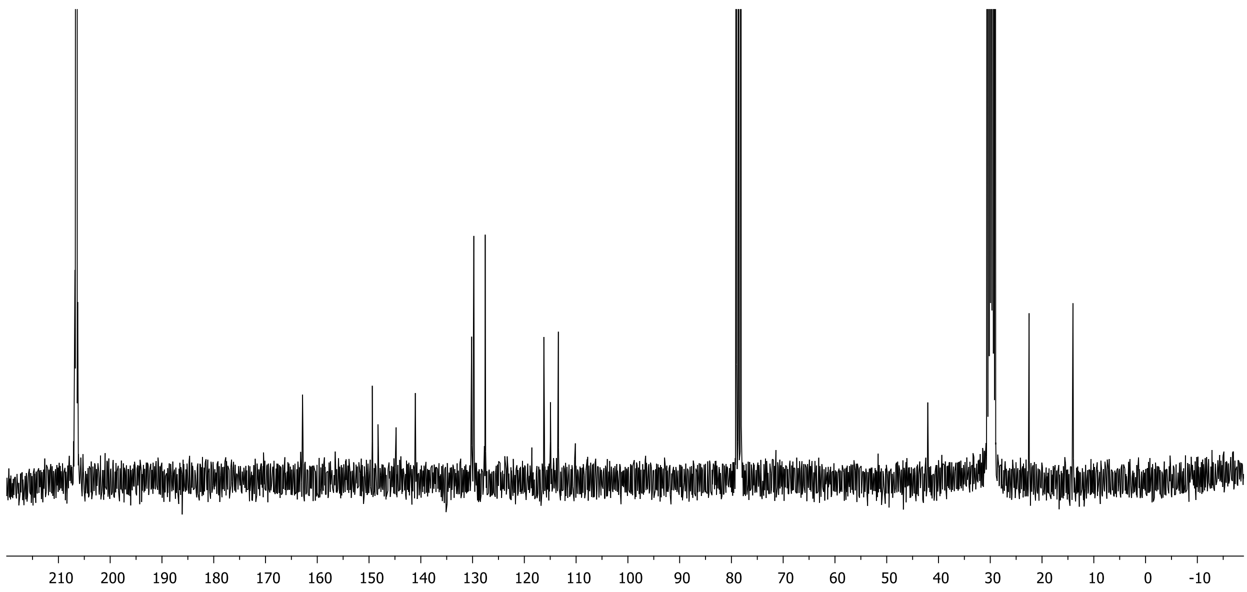


Absorption and emission spectra of 2c

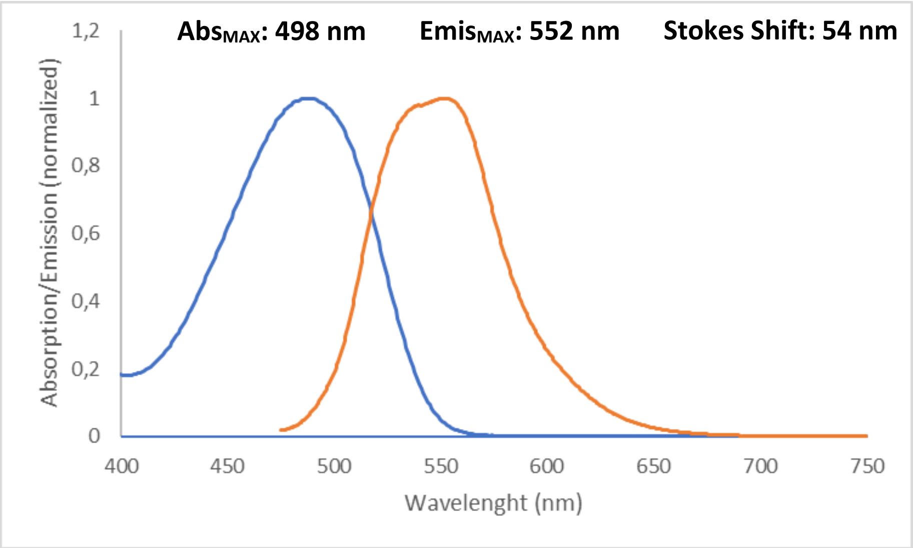

# Compound 2d

## $^{1}$H NMR spectrum of 2d

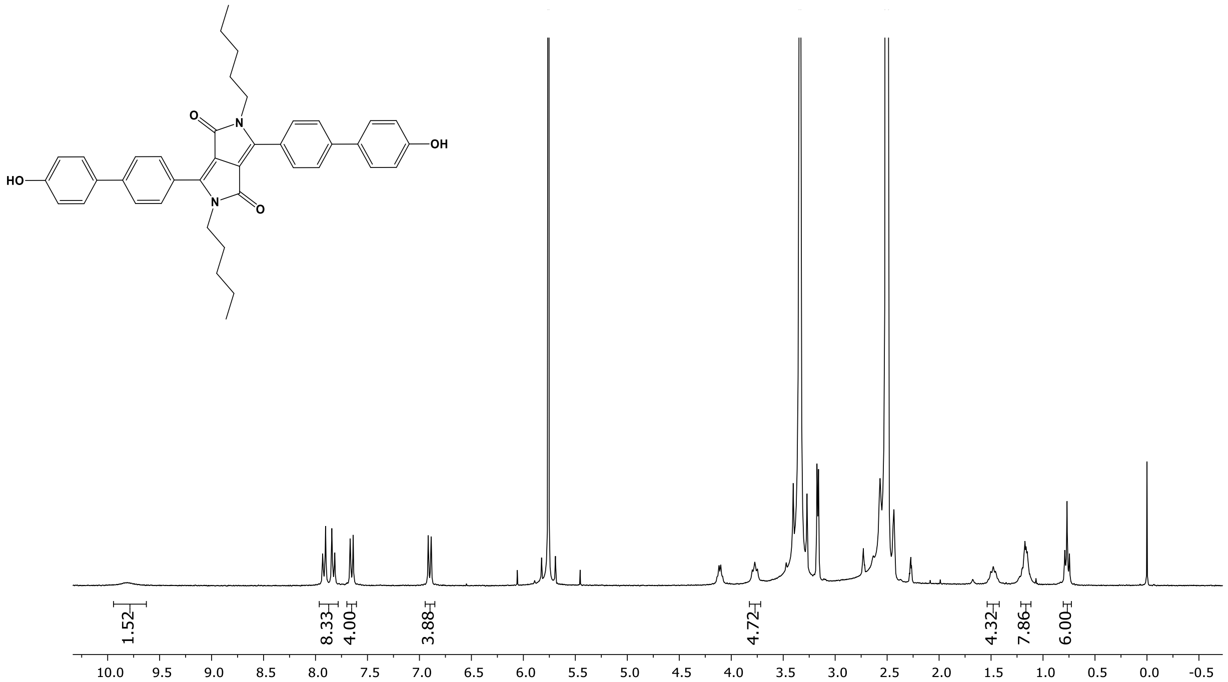


## $^{13}$C NMR spectrum of 2d

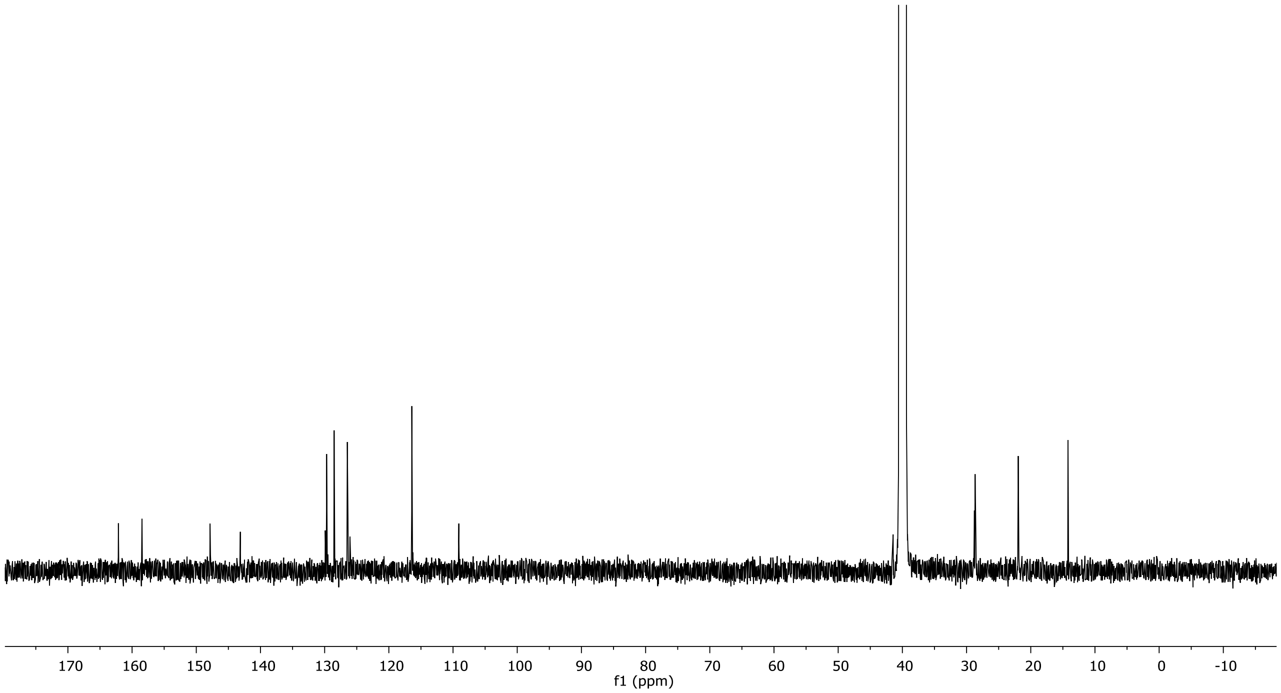

**Absorption and emission spectra of 2d**

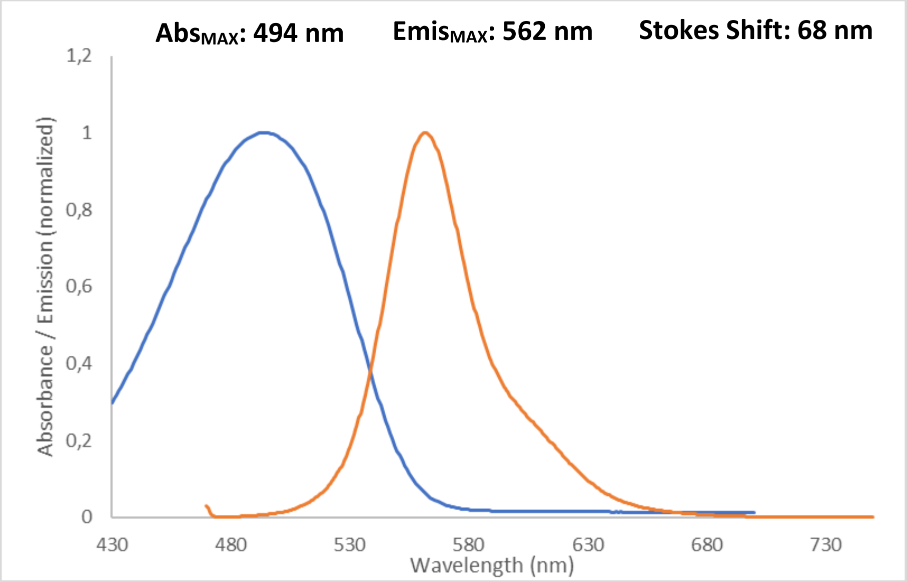

# Compound 2e

## $^{1}$H NMR spectrum of 2e

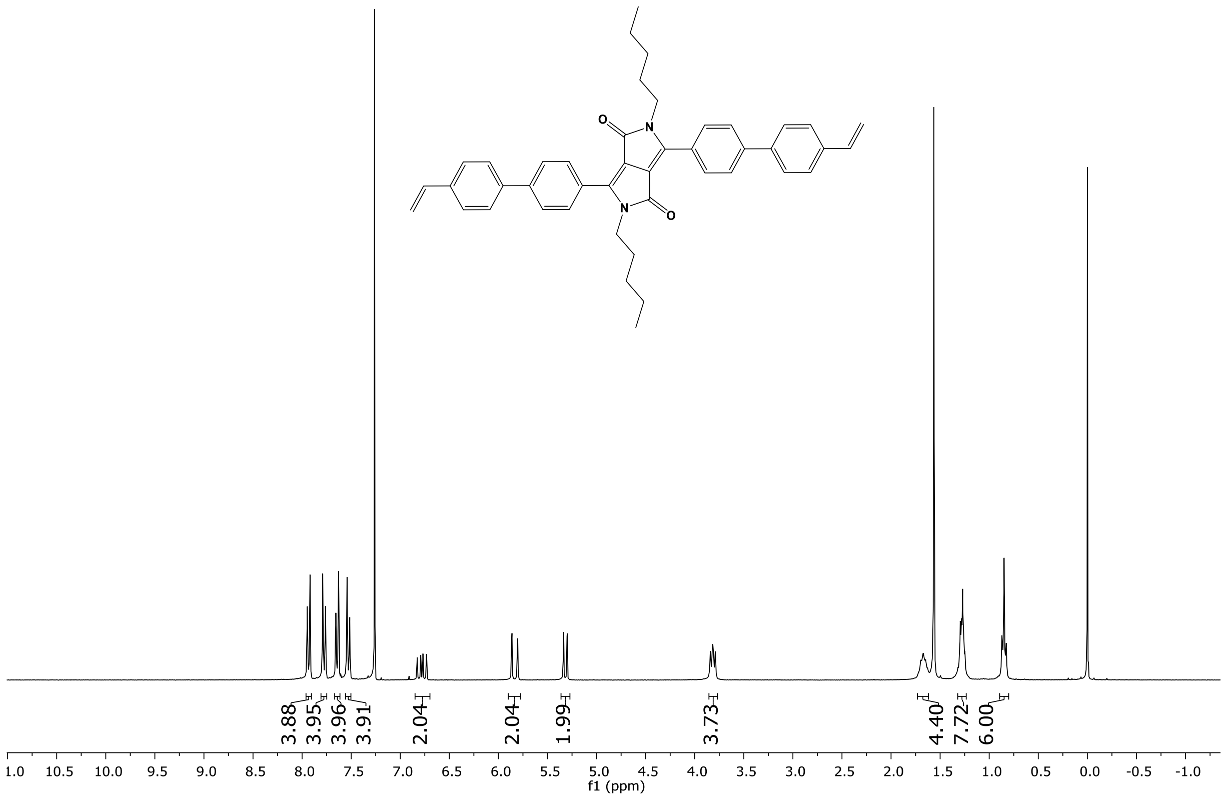


## $^{13}$C NMR spectrum of 2e

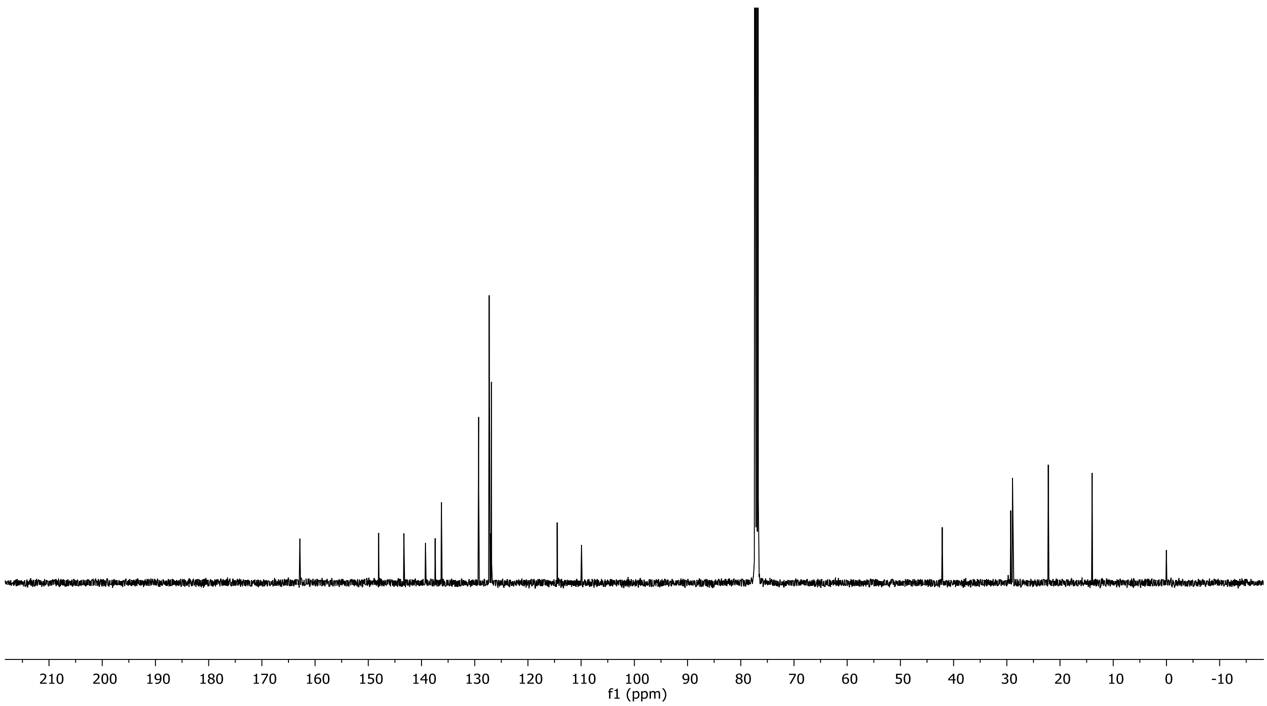

**Absorption and emission spectra of 2e**

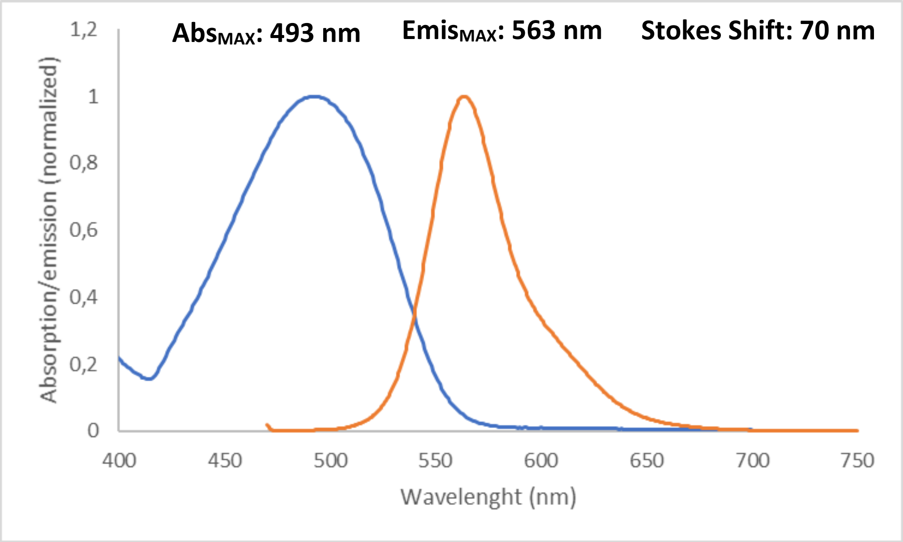

# Compound 2f

## $^{1}H$ NMR spectrum of 2f

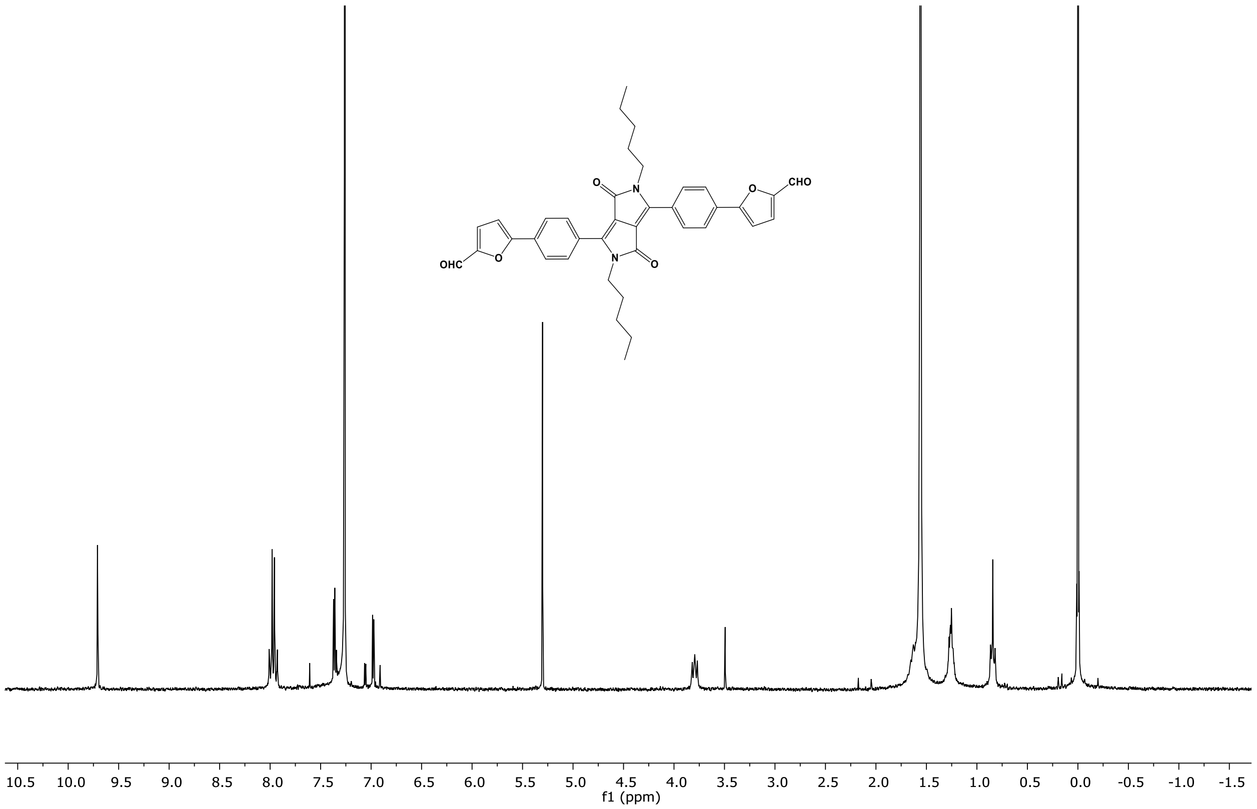


## $^{13}C$ NMR spectrum of 2f

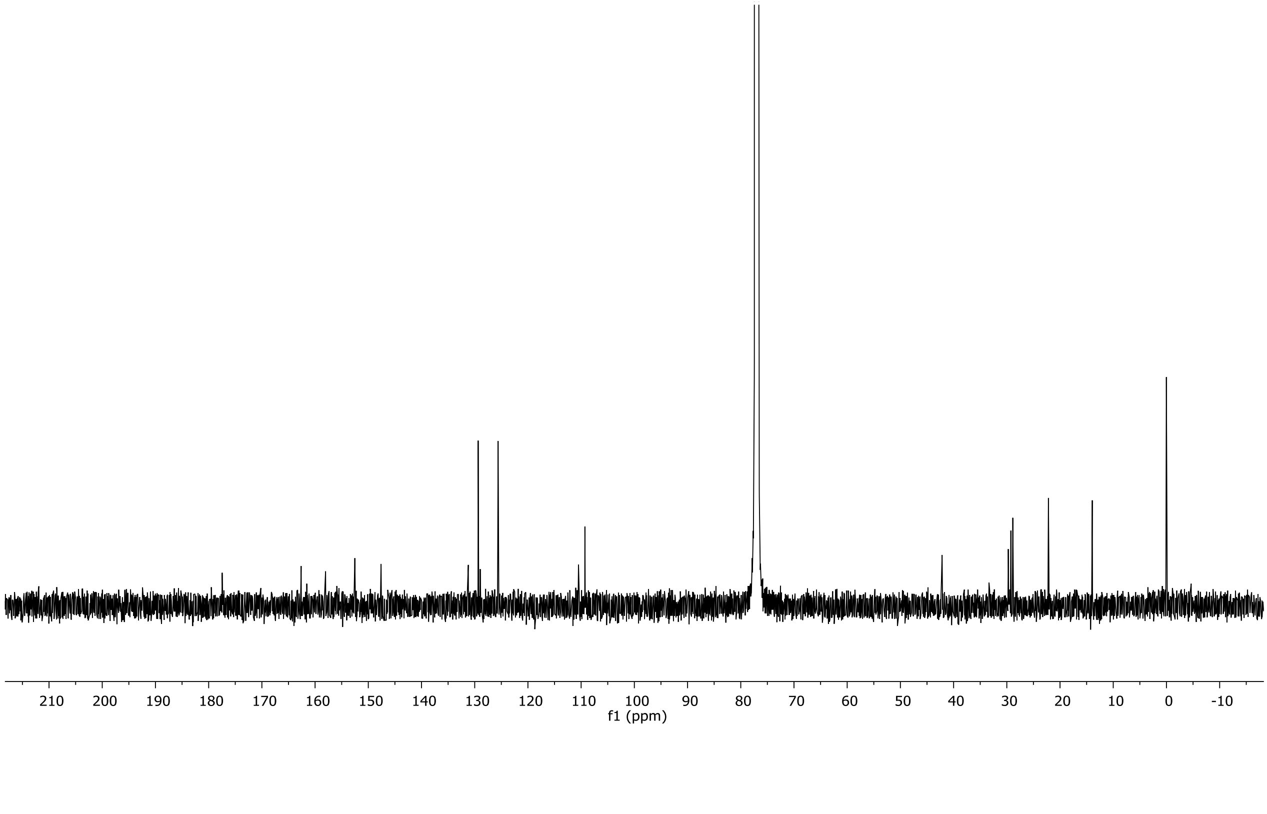

**Absorption and emission spectra of 2f**

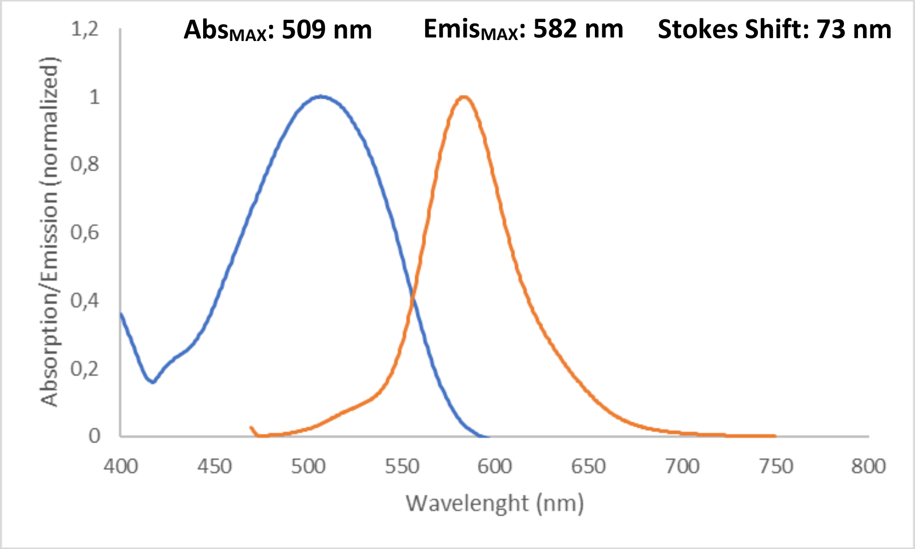

# Compound 2g

## ¹H NMR spectrum of 2g

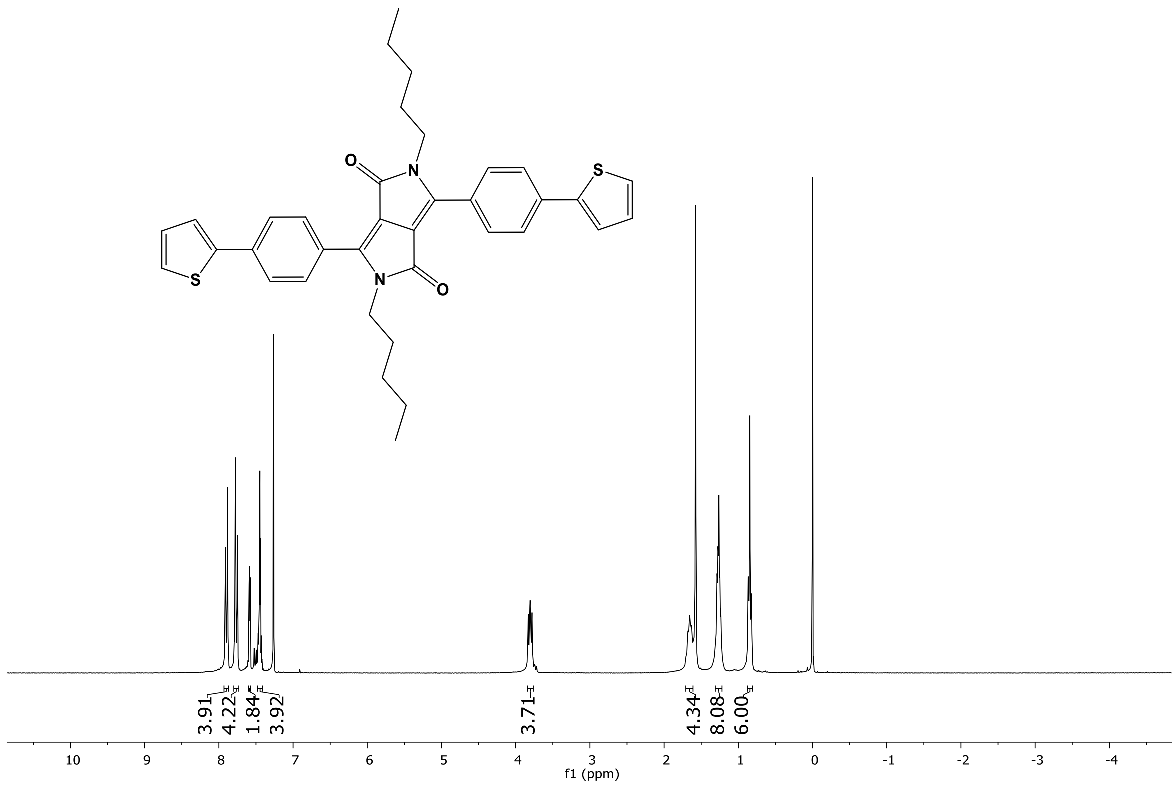


## ¹³C NMR spectrum of 2g

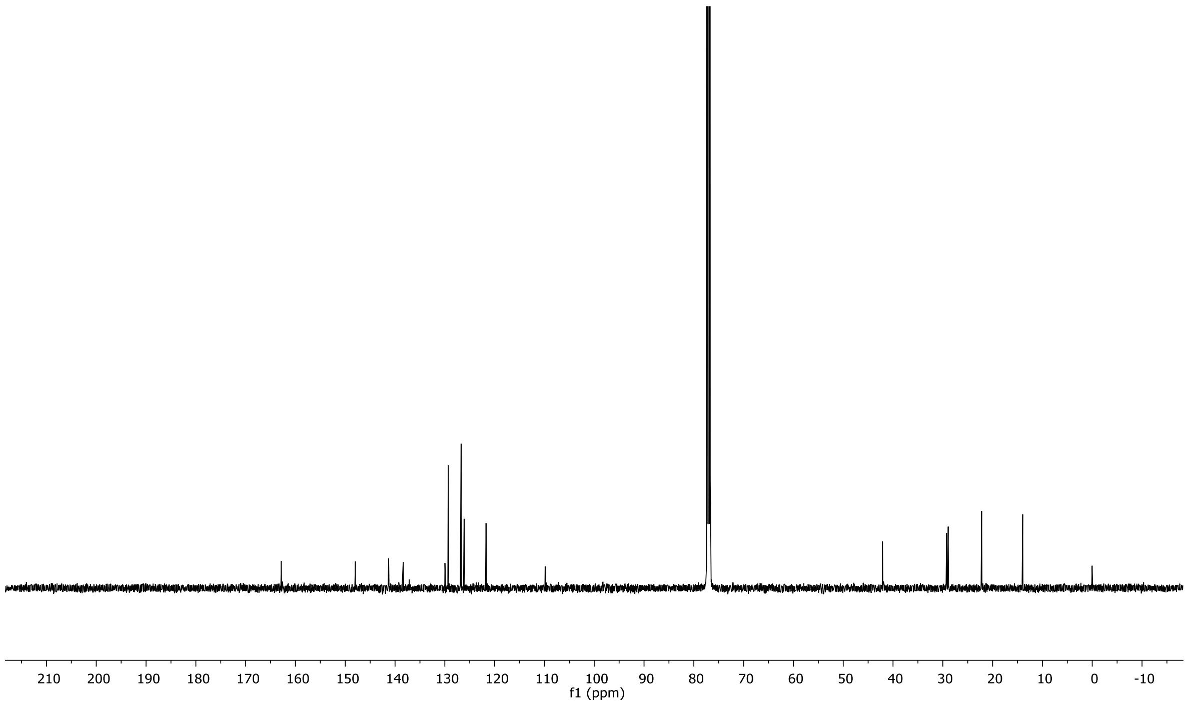

**Absorption and emission spectra of 2g**

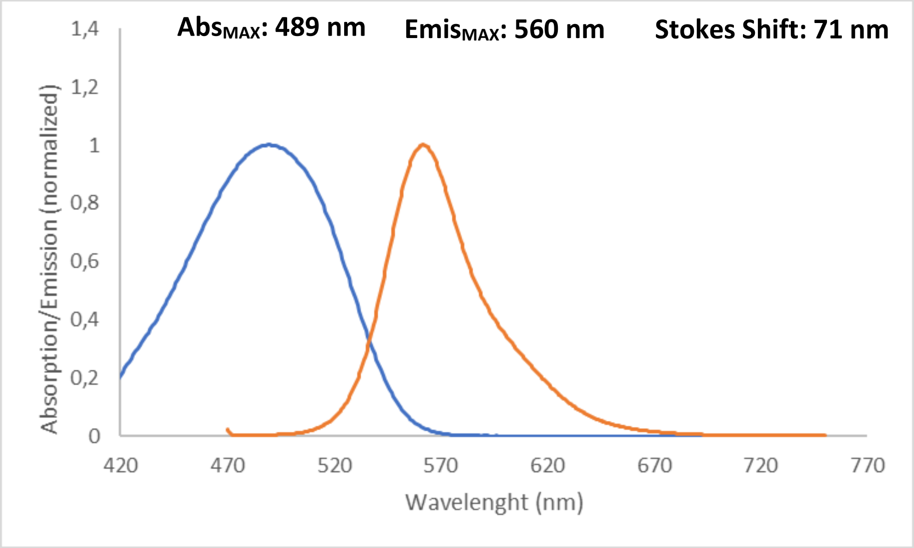

Supplement: Supplementary file 1 [file molecules-26-04758-s001.zip › molecules-1293767-supplementary.pdf]
